# Supplementary material for: Are carbon emissions trading and green financial instruments synergistic? -Comprehensive quantitative research based on content analysis
Source: PLoS One. 2024 Mar 7;19(3):e0298601. doi: 10.1371/journal.pone.0298601 (PMC10919601; doi:10.1371/journal.pone.0298601)
Supplement: S1 Data — (ZIP) [file pone.0298601.s001.zip › Data/地级市Z_F工作报告.zip!/╕╩╦α/╬Σ═■╩╨/└┤╫╘╣1⁄2╓┌║┼í╛┬φ┐╦╩2╛▌═°í┐_2012.docx]

各位代表：

　　现在，我代表市人民政府，向大会作工作报告，请予审议，并请各位政协委员和列席人员提出意见。

　　一、过去五年的主要工作

　　过去五年，全市各族人民在省委、省政府和市委的坚强领导下，坚持以科学发展观为指导，认真贯彻省委“四抓三支撑”的总体思路、区域发展战略和跨越式发展的总体要求，把石羊河流域重点治理作为首要任务，大力实施工业强市和生态安全战略，培育壮大支柱产业，推进金武一体化进程，开拓进取，扎实工作，经济快速增长，各项事业长足进步，民生有效改善，社会和谐稳定，本届政府确定的各项目标任务圆满完成。

　　——经济发展步伐不断加快，人民生活水平显著改善。过去五年，特别是去今两年，全市多数主要经济指标增速高于全省平均水平，固定资产投资、财政收入增长速度等指标居全省前列，是武威建设项目最多、投资额度最大、发展速度最快、综合效益最好的时期。今年，预计全市生产总值达到267.5亿元，比上年增长13.7%，是2006年的1.9倍，年递增11.9%。全社会固定资产投资303亿元，比上年增长60%，是2006年的3.9倍，年递增31.6%。工业增加值达到81亿元，比上年增长20.4%，是2006年的2.7倍，年递增18%。社会消费品零售总额达到90.3亿元，比上年增长19.8%，是2006年的2.2倍，年递增17.5%。大口径财政收入达到20亿元，比上年增长54.3%，是2006年的3.2倍，年递增25.9%。一般预算收入达到10亿元，比上年增长55.5%，是2006年的3.2倍，年递增26.1%。城镇居民人均可支配收入比上年增长15%，是2006年的1.8倍，年递增12.2%。农民人均纯收入比上年增长14%，是2006年的1.7倍，年递增11.3%。

　　——石羊河流域重点治理近期目标基本实现，生态环境恶化趋势得到遏制。2007年12月国家批准实施《石羊河流域重点治理规划》以来，全市累计关闭农业灌溉机井3318眼，压减农田灌溉配水面积64.25万亩，改建干支渠824.67公里，配套田间渠灌工程102.85万亩，配套管灌工程20.03万亩，新建大田滴灌工程31.14万亩。西营河专用输水渠建成投入运行。水权水价改革深入推进，节水型社会建设试点工作通过中期评估。经过五年特别是去今两年的艰苦努力，石羊河流域重点治理近期目标如期实现。蔡旗断面过水量去年超过2.6亿立方米，今年已达到2.72亿立方米，民勤盆地地下水开采量可控制在0.89亿立方米，全市水资源配置总量比2006年减少7.1亿立方米，地下水开采量削减6.1亿立方米。《石羊河流域重点治理调整实施方案》通过水利部审查，4个灌区节水改造工程开工建设。生态建设快速推进，今年完成人工造林33万亩、封山（沙）育林（草）31万亩，《石羊河流域防沙治沙及生态恢复规划》启动实施。民勤盆地干涸51年之久的青土湖连续两年人工形成了季节性水面，目前达到10平方公里，生态恶化趋势得到有效遏制，区域环境质量持续改善。

　　——工业和服务业加快发展，新型产业体系初步形成。累计完成工业固定资产投资273.24亿元，实施工业项目520项，其中亿元以上项目110项。工业园区建成区面积达到47平方公里，比2006年增加32平方公里，入驻企业352户。去年以来，强力推进工业强市战略，引进建设了一大批骨干项目，其中亿元以上项目86项，工业经济进入了加速发展阶段。能源工业发展提速，建成了红沙岗一号矿井及金阿铁路专用线、天祝华祥3万吨页岩油等项目，酒钢天威200万吨洗精煤项目开工建设，风电、光电开工装机规模分别达到198兆瓦和98兆瓦。化学工业产业链不断延伸，建成了鑫淼4万吨石灰氮等项目，新开工荣华易地搬迁技改和鑫淼、酒钢天威、荣达各60万吨电石等项目。装备制造业起步良好，建成了八冶武威10万吨钢结构等项目，新开工普信电器100万台冰箱冷柜、甘肃格林西北电动车生产基地等项目。材料工业规模扩大，天祝碳基材料产业基地加快建设，古浪祁连山、天祝神鹰、民勤荣达800万吨新型干法水泥等项目开工建设。食品工业改造升级步伐加快，青啤武威公司60万千升啤酒、威龙10万吨葡萄酒堡等项目有序推进。现代物流业逐步兴起，黄羊众远公铁联运物流中心、武威综合物流园区等项目开工建设；累计投入资金10.2亿元，新建和改造提升各类市场146个。旅游产业发展加快，预计今年全市接待国内外游客250.2万人次，增长39.6%，实现旅游总收入11.47亿元，增长38.9%。

　　——“三农”工作不断强化，农业农村经济稳步发展。大力发展节水高效农业，促进了农民收入持续增长。以“设施农牧业+特色林果业”为主攻方向，加快调整农业结构。去年以来，建成设施农业18万亩，累计达到31万亩；建成特色林果业18万亩，累计达到52.8万亩。推广大田高效节水面积103.6万亩、旱作农业50.7万亩。畜牧业增加值占农业增加值的比重达到31%，高于全省7.4个百分点。培育省级以上产业化龙头企业32家、农民专业合作社704个。累计输转城乡劳动力194.48万人次，实现劳务收入136.41亿元。改造农村危房5.5万户，建成农村户用沼气4.7万户，解决了62万人的饮水安全问题。贫困人口比2006年减少10.32万人，贫困面下降了5个百分点。

　　——基础设施建设力度加大，固定资产投资大幅增长。加大项目争取和实施力度，共实施基础设施建设项目2211项，其中亿元以上项目164项，累计完成固定资产投资820亿元。去今两年，完成固定资产投资492.17亿元，占到五年总和的60%。金大快速通道路基全线拉通，铺油通车13公里，完成通道绿化98.94公里。金武、双营、永古高速和武西高等级公路建设进展顺利，在建高等级公路里程达到412公里。新建改造农村公路4482.2公里，全市公路通车里程达到8851.2公里，路网密度达到26.7公里/百平方公里。完成红崖山、张义、安远等灌区续建配套与节水改造工程，实施了曹家湖、十八里堡、龙潭河、鞍子山水库除险加固工程，治理水土流失面积1761平方公里。实施城区配网、西部农网完善等项目，供电保障能力不断增强。中心城市规模扩大，建成区面积由2006年的24.6平方公里扩大到30平方公里，城区人口由26万人增加到41万人。新建改造城区道路51公里、供排水管网74公里。火车站站房改扩建、城北和城南集中供热等一批重点项目建成运营。去年，新城区建设全面启动，体育馆、博物馆、影剧院、天马湖景观治理工程、热电联产及公共卫生综合服务楼、水务公共服务楼等重点项目开工建设。县城和重点小城镇综合承载能力不断增强。

　　——民生保障水平逐步提高，社会各项事业协调发展。千方百计扩大就业，城镇新增就业5.8万人，城镇登记失业率控制在4%以内，解决高校毕业生就业1.43万人。基本养老保险、医疗保险实现全覆盖，村干部、被征地农民养老保险等制度逐步建立健全。低保标准逐年提高，覆盖面不断扩大，城乡低保对象分别达到5.26万人和20.41万人。累计建成廉租房和经济适用房12558套、66.5万平方米，新建改建敬老院16所。新建、改扩建校舍79.3万平方米，消除危房46.1万平方米，城乡免费义务教育基本实现，“两基”国检责任目标基本完成。去年以来，新办了兰州交通大学新能源与动力工程学院和甘肃威龙葡萄酒业专修学院，今年实现首批招生。武威职业学院成功创建为省级示范性高职院校，纳入“国家示范性高等职业院校建设”计划，武威卫校整建制并入武威职业学院，开启了武威发展高等教育的新阶段。科技创新能力增强，科技对经济增长的贡献率达到45%。公共卫生体系和突发公共卫生事件应急机制逐步建立，基层医疗卫生服务体系建设加快，基本药物零差率销售覆盖基层医疗服务机构，中医药特色医疗服务全面推行，新农合参合率达到95%以上。低生育水平保持稳定，人口出生率控制在了9‰以内。文化体育设施不断完善，建成乡镇综合文化站61个、农家书屋1125个，建成体育场地33.6万平方米。市（区）县全部被命名为省级“双拥模范城（县）”。加强监督检查，安全生产责任制有效落实。强化食品药品安全监管，保障了群众饮食用药安全。加快藏区经济社会发展，保持了民族团结、宗教和顺。“五五”普法规划全面完成，信访、维稳工作不断加强，平安武威创建活动深入推进。统计、气象、消防、人防、档案、老龄、助残、地方志、外事侨务、防震减灾等各项事业取得新成就。坚持重承诺、办实事，一批关系群众切身利益的实际问题得到妥善解决。 去今两年两次提高机关事业人员津补贴标准，工资水平基本达到全省市州平均水平。今年为民办理的10件实事基本实现。

　　——改革开放深入推进，发展环境不断优化。土地经营权流转制度基本建立，国有企业改革、集体林权制度主体改革、粮食流通体制改革任务全面完成。医药卫生体制改革、财政管理体制改革、供销体制改革深入推进。加大人事制度改革力度，引进急需紧缺人才526名。大力推进诚信武威建设，积极搭建政府信用平台，组建国有资产投资公司，设立了8家中小企业担保公司。今年，全市金融机构存款余额预计达380亿元、贷款余额达205亿元，分别比上年增长17.64%和27.32%。农村金融改革取得阶段性成果，累计发放妇女小额担保贷款22.4亿元。对内对外开放进一步扩大，去今两年招商引资实际到位资金122亿元，占五年总和177亿元的69%。外贸进出口总额五年累计达到4409万美元，年均增长26.89%。

　　——依法行政水平不断提高，政府自身建设进一步加强。健全完善政府议事决策规则和信息发布制度，对涉及群众重大利益的社会事项实行听证、公示和专家咨询制度，政府决策的民主化、科学化水平不断提高。自觉接受人大法律监督和政协民主监督，认真办理人大代表议案和政协委员提案。加快政府职能转变，深入推进政府机构改革，规范和减少行政审批收费事项，扎实开展“创先争优”、“作风建设年”、“提质增效年”等主题活动，推行行政问责制，推进政务公开，行政效能不断提高。严格落实党风廉政建设责任制，加强行政监察和审计监督，严肃查处了一批违纪违法案件。

　　各位代表！过去五年，全市改革发展的成绩有目共睹，来之不易。这是省委、省政府和省直各部门真诚关心、大力支持的结果，是市委统揽全局、正确领导的结果，是市人大、市政协和社会各界监督支持的结果，是全市各族人民共同努力的结果。在这里，我谨代表市人民政府，向全市各族人民，向各民主党派、工商联、人民团体和社会各界人士、离退休老同志，向驻武各部队、武警官兵、民兵预备役人员和中央、省属驻武各单位，以及所有关心支持武威改革发展的同志们、朋友们，表示诚挚的感谢和崇高的敬意！

　　过去五年，全市改革发展的实践，使我们对市情特征和经济社会发展规律有了新的认识。我们深切地体会到，推动跨越式发展，必须坚持党的领导，全面贯彻落实中央和省委、省政府及市委的各项决策部署，解放思想，更新观念，抢抓机遇，狠抓落实，大力提高贯彻力和执行力;必须坚持发展第一要务，从武威的实际出发，抓发展、抓项目、抓投资，以发展凝聚人心、用项目衡量政绩，培育支柱产业，促进城乡融合，推动工业强市，扩大经济总量，夯实发展基础；必须坚持不懈推进生态建设，统筹处理好经济社会发展与生态保护的关系，建设节水型社会，破解资源环境制约，增强可持续发展能力；必须坚持保障和改善民生，认真解决关系群众切身利益的实际问题，不断提高人民群众生活水平，为人民群众谋福祉、办实事；必须坚持加强作风建设，大力弘扬甘肃精神，正视差距、奋起直追，治庸治懒、提质增效，求真务实、埋头苦干，廉洁奉公、干净干事，营造良好的发展环境。

　　回顾过去，我们也认识到，实现跨越式发展，还面临诸多困难和问题，制约发展的因素还很多。在全省加快发展的大格局中，武威整体发展水平低，改革发展任务艰巨繁重。经济总量小、实力弱，人均水平低，财政困难；经济结构层次低，工业处于低水平发展阶段，重大项目和骨干企业少；自然条件严酷、生态环境脆弱、水资源严重匮乏的状况没有根本改变，石羊河流域治理任务长期而艰巨；城镇化水平低，带动农村能力弱，农村基础条件差、贫困面大、贫困人口多，保障改善民生任重道远；部分干部思想观念、工作作风还不适应新形势新任务的要求，依法行政水平和行政效能有待提高，经济社会发展的环境还需进一步优化。对于这些困难和问题，我们一定高度重视，认真对待，下决心解决。

　　二、今后五年的总体思路、奋斗目标和重点工作

　　今后五年，是我市转变发展方式、夯实发展基础、提升发展实力、推动跨越发展的重要机遇期。国家出台了实施新一轮西部大开发战略、支持甘肃发展和藏区发展等重大政策，继续支持石羊河流域重点治理、防沙治沙和生态恢复建设，省委、省政府深入实施区域发展总体战略、循环经济和金武一体化规划，为我市提供了难得的发展机遇。特别是省政府批准实施《甘肃省统筹城乡发展试验区武威城乡融合发展核心区总体规划》，更为我们推进跨越式发展提供了特殊的政策支持。东部沿海地区资源性产业和高载能产业向西部地区加速转移，有利于我们发挥区位、土地、光热、风能和农产品资源丰富、人力资源富余的比较优势，更好地招商引资。近两年开工建设的一批重大工业、能源、交通、水利项目，为今后发展奠定了良好的基础。我们必须进一步解放思想，抢抓机遇，只争朝夕，埋头苦干，推动跨越式发展。

　　刚刚闭幕的市第三次党代会科学规划了今后五年的宏伟蓝图，指明了发展的方向和路径。政府工作的总体思路是：以邓小平理论、“三个代表”重要思想为指导，深入贯彻落实科学发展观，按照省委区域发展战略和跨越式发展总体要求，全面落实市第三次党代会精神，坚持以发展凝聚人心、用项目衡量政绩，大力实施城乡融合、工业强市和生态立市战略，培育壮大支柱产业，推进石羊河流域重点治理，加强基础设施和社会事业建设，促进金武一体化进程，提高民生保障水平，维护社会和谐稳定，奋力开创全市经济社会跨越式发展的新局面。

　　今后五年，经济社会发展的主要奋斗目标是：经济总量保持两位数增速，主要经济指标比2011年翻一番、增速高于全省平均水平。2016年，全市生产总值达到600亿元，年递增14%；工业增加值达到300亿元，年递增25%；全社会固定资产投资五年总计达到2000亿元；大口径财政收入达到50亿元、一般预算收入达到25亿元，年递增均为20%；社会消费品零售总额达到200亿元，年递增17.2%；城镇居民人均可支配收入和农民人均纯收入分别达到24100元、10000元，年分别递增13%、14%。努力把武威建成全省重要的能源化工、制造业和农产品基地，西北重要的综合交通枢纽和物流节点区，全国节水型社会示范区和国家级生态恢复示范区。

　　围绕上述总体思路和奋斗目标，重点把握好七个方面的工作。

　　一是竭力实施城乡融合战略，加快城乡一体化进程。全力以赴实施《武威城乡融合发展核心区总体规划》，建成金大快速通道，构建核心区脊椎和主动脉，建设通组团和园区、通乡镇和旅游景区、通村组的三级干支公路网络，衔接高速公路、铁路、航运，形成承东启西、接南连北的交通枢纽，实现空间融合。以凉州城区、大靖新城和永丰、黄羊、土门三个组团核为中心，加快教育、卫生、科技、文化、体育等社会事业发展和城镇基础设施建设，促进公共资源均等共享，形成中小城镇集群，提高城镇化水平。以工业园区和物流中心为载体，加快特色产业聚集，建设工业集群，提高核心区工业化水平。以绿洲区域为基地，建设设施农牧业集群，发展现代农业。以“一个生态绿轴、六条水源涵养廊道、多个生态协调区”为主体功能区，造林绿化、防沙治沙，构建城乡核心区生态安全体系。以提升居民素质为目标，推进城乡文化一体化建设，探索建立新型社会管理体系，创建精神文明示范区。积极推进天祝县融入兰白经济圈，以金强河道综合治理开发为重点，建设金强川经济综合发展核心区，带动藏区跨越式发展。以民勤县城和红沙岗为中心，围绕民武路、民昌路建设核心经济区，促使产业、人口向城镇、工业区和绿洲片带集中，实现率先发展。积极实施《金武区域经济一体化发展规划》，加强与金昌市的产业、技术协作，形成金昌国家级经济技术开发区与我市工业园区“一区多园”建设格局，推进金武一体化进程。

　　二是全力实施工业强市战略，培育壮大支柱产业。坚持引项目、找矿藏、建园区、上规模、增效益，构建具有比较优势的新型工业化发展体系。大力发展能源工业，重点建设煤炭和风电、光电等能源项目。加快发展化工、冶金、碳基材料、电石和建材等项目，延伸产业链。积极发展车辆制造、家电、光伏组件、机械制造等项目，建设制造业基地。改造提升食品工业，重点发展以啤酒、葡萄酒为主的酿造业，肉奶、果蔬和休闲保健食品加工业。创新园区管理体制和运行机制，加大基础设施建设投入力度，启动建设大靖工业园区、满家滩工业园区，提升园区建设水平。全方位开展招商引资，积极引强入武。落实优惠政策，支持中小企业和民营经济做大做强。发展循环经济，推进节能减排。培育壮大现代物流业，抓好重大物流项目建设，加大市场建设力度，构建现代商贸流通体系。加快发展特色旅游业，完善旅游规划体系，深度挖掘文化旅游资源，打造历史文化游和生态观光游等特色旅游品牌。加强旅游基础设施和人才队伍建设，加大宣传推介力度，强化旅游市场监管，提升旅游业服务水平。加大工业化和信息化融合力度，加强农业信息化产品推广，推进“数字城市”建设，提高全社会信息化水平。

　　三是大力实施生态立市战略，促进生态环境持续好转。着力破解“结构性缺水”命题，全面推进节水型社会建设。坚持“南护水源、中调结构、北治风沙”的方针，统筹抓好“节水、治沙、造林、防污”四个重点，探索建立“国家有投入、科技做支撑、农民有收益”的生态建设长效机制。全面落实重点治理措施，提前五年实现石羊河流域重点治理远期目标，到2016年，民勤蔡旗断面下泄水量达到2.9亿立方米以上、民勤盆地地下水开采量减少到0.86亿立方米，全市年用水量控制在16.46亿立方米以内，地下水年开采量控制在4.84亿立方米以内，森林覆盖率提高到18%。全面完成《石羊河流域防沙治沙及生态恢复规划》建设任务，推进天龙百万亩生态林建设项目，深化农村集体林权制度配套改革，将治沙生态林承包给农民管护经营，完成国有林场改革。统筹抓好生态经济型防护林网、南部祁连山水源涵养区建设与保护，严格落实禁止开荒、禁止打井、禁止放牧、禁止乱采滥伐的规定，加大湿地保护恢复和建设力度，提高生态系统自我恢复功能。加强工业点源、农业面源和生活污染综合治理，有效控制污染物排放，确保环境安全。

　　四是加大农业结构调整力度，加快推进新农村建设。突出调结构、修公路、兴水利、建新房、抓扶贫、促增收，扎实推进新农村建设。大力推广“设施农牧业+特色林果业”的主体生产模式，到2016年，以日光温室为主的瓜菜业达到20万亩；以养殖暖棚为主的畜牧业达到30万亩；以葡萄、红枣、优质梨、枸杞为主的特色林果业达到100万亩以上；建设灌区节水高效农业、山区旱作农业现代农业区，力争实现户均2亩棚、2亩经济林和人均1亩节水高效大田、农民人均纯收入翻一番的目标。完善村镇和村庄规划，改造农村危房20万户。全面解决农村饮水安全问题，加快沼气工程建设，美化农村居住环境。深化农村综合配套改革，积极创建省级示范县和市级示范村镇。加快农业科技成果转化应用，加大投入力度，大力引进、推广农业新材料、新设备、新技术、新品种，提高农业机械化水平。加强农民实用技术培训，每年培训农民50万人次。统筹抓好龙头企业与原料基地、生产加工与市场流通体系建设，推进标准化生产，提升农业产业化经营水平。加大扶贫开发和生态移民力度，抓好海拔2800米左右高深山区农牧民“下山入川”工程，力争完成移民7万人。

　　五是加强基础设施建设，不断增强发展后劲。以交通、水利、电力、城镇建设为重点，完善基础设施布局，为经济社会发展提供有力支撑。加强交通运输设施建设，加快实现省、市级通道高速化，建成金武、双营、永古高速公路，开工建设武民高速、华藏寺至天堂寺公路、民勤县城至红沙岗一级公路，实施民调渠沿线防沙治沙旅游公路项目，积极创造条件，在沿线建设枸杞等生态经济林带。升级改造县、乡公路，乡镇、村通公路率均达到100%，通油路率达到100%和95%。到2016年，全市公路总里程达到1.5万公里，路网密度达到45.3公里/百平方公里。争取增建干塘至武威铁路二线、兰州至张掖高速铁路中川至武威段。加快农田水利基本建设，实施小型农田水利重点县、专项县建设项目，全面完成灌区末级渠系配套建设。建成杂木河毛藏寺水库及杂木河供水工程、石门河引水和大通河防洪等工程。完成红崖山等水库清淤和杂木河等重点河道整治，增强石羊河流域水资源调蓄能力。加快电网设施建设，坚持负荷增长与电网建设、电源建设与电网建设同步推进，新建一批输变电工程，实施配电网络改造，提升工业园区、重点企业、城市和设施农业用电保障能力。坚持规划、建设、经营、管理并重，加快新城区建设，抓好老城区改造，完善供热、供气、供排水、环卫等市政设施，三年基本完成棚户区改造，建设生态宜居城市。推进县城和小城镇基础设施建设，完善配套服务功能。

　　六是统筹发展社会事业，提高民生保障水平。按照普惠制和尽力而为、量力而行的原则，集中力量每年办一批改善民生的实事。优先发展教育，加强教育基础设施建设，合理配置教育资源，优化布局结构，实现教育公平。不断改善高等教育办学条件，抓好武威职业学院国家级示范高职院校、民勤教育城等项目建设，着力打造凉州城区和黄羊两个职业教育园区。全市高中教育、特殊教育达到全省平均水平，职业教育、学前教育、义务教育、民族教育和民勤县总体办学指标达到全省先进水平，基本普及14年基础教育，进入全省教育强市行列。深化医药卫生体制改革，完善覆盖城乡的基本医疗卫生服务体系、基本医疗保障体系和基本药物保障供应体系。加大统筹解决人口问题的力度，稳定低生育水平。深入推进文化体制改革，促进文化大繁荣大发展。强化社会主义核心价值体系教育，大力弘扬诚信友爱、敬老爱老、公平正义的社会风尚。加强公共文体设施建设，构建公共文化服务体系和现代传播体系，大力发展文化产业，完成文艺院团改制任务。千方百计扩大就业，坚持以创业带动就业，加大政策扶持力度，新增城镇就业6万人。继续抓好劳务输转，提高输转质量和效益。加大保障性住房建设力度，满足困难群众基本住房需求。完善最低生活保障和养老、失业、工伤、生育等保险制度，提高城乡社会保障和救助能力。

　　七是加强和创新社会管理，维护社会和谐稳定。坚持用群众工作统揽信访工作，进一步畅通和规范群众诉求表达、利益协调、权益保障渠道，加大矛盾纠纷摸排和调处力度，积极预防和妥善处理各类群体性事件。加强公民意识和社会责任意识教育，做好流动人口服务管理和特殊人群帮教转化工作，预防和减少社会矛盾。扎实推进平安武威创建活动，加强社会管理综合治理，严厉打击各类违法犯罪活动，增强人民群众安全感。加强应急管理体系建设，健全完善各类应急预案，加快应急物资储备库、应急避难场所建设，提高综合防范和抵御灾害能力。强化食品药品安全监管，保障人民群众身体健康和生命安全。严格落实安全生产责任制，严防重特大事故发生。加快民族地区经济社会发展，维护藏区稳定，促进宗教和顺。加强社区建设，提高社区服务质量。深入开展“六五”普法，加快依法治市进程。完善政府调控和市场调节机制，加强价格监管，保持物价总水平基本稳定。

　　三、2012年的主要工作

　　明年，经济社会发展的主要预期目标是：生产总值达到315亿元，增长14%；工业增加值达到106亿元，增长25%；全社会固定资产投资达到403亿元，增长33%；大口径财政收入达到26亿元，增长30%；一般预算收入达到13亿元，增长30%；社会消费品零售总额达到106亿元，增长17.5%；城镇居民人均可支配收入增长13%；农民人均纯收入增长14%。城镇登记失业率控制在4%以内，人口自然增长率控制在7‰以内，居民消费价格指数涨幅控制在4.8%以内，单位生产总值能耗和主要污染物排放完成省上下达的控制指标。

　　重点做好八个方面的工作：

　　（一）以城乡融合发展核心区建设为重点，加快城乡融合发展步伐。全力实施《武威城乡融合发展核心区总体规划》。加大融资力度，创新融资方式，多渠道筹集资金，加快金大快速通道建设，力争实现全线铺油通车。建设一批通园区、通景区、通乡镇、通村组的公路项目，启动实施沿线公交站点、油气站点、物流节点等配套工程，不断培育轴线带动能力。围绕核心区总体规划，修编武威市城市总体规划，编制大靖城总体规划，完善永丰、黄羊、土门三个组团基础设施、产业发展、公共服务相配套的发展规划，分步做好核心区村镇规划，科学谋划教育、卫生、文化、体育、旅游等公共服务设施布局。加强政策和项目对接，加大资金整合力度，力争新开工一批项目。高标准完成金大快速通道主线和支线绿化任务，在通道两侧建立50至100米以黄冠梨为主的特色林果示范长廊。打破行政和园区界限，探索灵活多样的园区和村镇建设模式，重点建设8个千亩设施农业示范区、9个标准化规模养殖示范区、10个生态经济林示范区、1.3万亩节水灌溉示范基地，率先建成一批示范区和样板村镇。抓好天祝县金强川综合发展核心区和民勤县核心经济区规划编制工作，加快产业培育和基础设施配套建设步伐。加强区域协作，着力推进金武一体化进程，抓好同金昌市、金川公司、酒钢公司的合作项目建设。

　　（二）以骨干项目和园区建设为重点，强力推进工业经济快速发展。狠抓重点工业项目建设。开工建设西班牙阿特萨100兆瓦光伏组件、江西赛维光伏产业园、泰康制药搬迁扩建等120个项目；加快推进酒钢天威和荣达公司各60万吨电石、山东泰安新能源重型车、湘电风能200台套风机、青啤武威公司60万千升啤酒等130个项目建设进度；争取建成荣华易地搬迁技改、中电国际20兆瓦和华电10兆瓦光电、响水煤业300万吨洗精煤、圣合机械3万辆电动车、古浪祁连山和天祝神鹰新型干法水泥等100个项目；重点做好2×1000兆瓦调峰火电、天祝2×300兆瓦煤矸石电厂、臭牛沟矿区煤炭勘探开采等重点项目前期工作。加快园区基础设施建设步伐。建成园区道路120公里、给排水管网110公里、供电线路56公里，开工建设新能源装备制造产业园等园区污水处理厂，完成园区基础设施投资8亿元以上；加大招商引资力度，完善激励机制，落实招商引资优惠政策，千方百计引强入武，力争签约招商引资项目150项以上，落实到位资金100亿元。强力推进节能减排，抓好黄羊工业园区国家循环化改造示范试点建设，加快推广循环经济模式，加大企业内部清洁生产审核力度，加强高耗能、高污染项目审批管理，坚决淘汰落后生产能力。加强运行监测分析，强化煤电油运和资金等要素协调服务，保障重点项目用地、用水、用电需求，确保工业经济快速增长。

　　（三）以节水、治沙、造林、防污为重点，加强生态保护与建设。落实《石羊河流域重点治理调整实施方案》建设任务，全面完成昌宁、黄羊、古浪、古丰灌区节水改造工程，做好生态移民试点工作，开工建设景电二期向民勤调水渠延伸工程。大力实施人工影响天气工程。加强水资源调度管理，确保平水年蔡旗断面过水量达到2.5亿立方米以上，重点治理区地下水开采量控制在4.95亿立方米以内。加快行业用水结构调整，大力推广高效节水技术。认真实施《石羊河流域防沙治沙及生态恢复规划》和天龙百万亩生态林建设项目，以民勤西线、北线，凉州、古浪民调渠沿线为重点，加大防沙治沙力度，完成治沙11.2万亩。认真执行国家草原生态补助奖励等政策，严格落实“四禁”规定，抓好南部祁连山水源涵养林建设与保护。加大湿地保护恢复和建设力度。推进退耕还林、退牧还草、天然林保护、“三北五期”、国家级公益林保护等项目建设，完成人工造林36万亩、封山（沙）育林（草）35万亩、义务植树1000万株、通道绿化1000公里，治理水土流失面积30平方公里。加强污染综合治理，强化水污染、工业固废污染防治和噪声整治，实施60个“以奖促治”农村环境连片整治项目、19个规模化畜禽养殖污染防治项目，提高废旧农膜回收率，推行农村垃圾、污水集中收集处理，减少农业面源污染。

　　（四）以设施农牧业和特色林果业为重点，加大农业结构调整力度。坚定不移地走“设施农牧业+特色林果业”的路子，新建设施农业6万亩、标准化养殖小区（场）180个，新增规模养殖户1万户，新建以葡萄、红枣、优质梨、枸杞为重点的特色林果基地20万亩，推广以垄膜沟灌、膜下滴灌等为主的高效节水技术142万亩、旱作农业60万亩。建设现代农业示范工程与科技支撑发展体系，加快推进农业机械化，促进农机农艺融合。加强农产品质量安全监测，大力发展无公害农产品、绿色食品和有机食品。扶持壮大农业产业化龙头企业，大力培育农民专业合作社和各类社会化服务组织，推进农产品生产加工流通一体化经营。整合教育培训资源，建立综合性劳动力培训基地，规范劳务市场管理，打造劳务品牌，完成劳务输转40万人次，实现劳务收入40亿元。抓好新农村试点县和20个市级新农村示范村建设，改造农村危房2万户以上，加快饮水安全和户用沼气等工程建设。启动实施“拆墙透绿”工程，搞好环境绿化美化。推进张义哈溪省级扶贫开发试验示范区建设，加大祁连山北坡浅山干旱地区、北部沿沙地带等特困片带扶贫攻坚力度，加快古浪县黄花滩灌区骨干水利工程建设，实施整村推进项目16个，不断改善特困片带群众生产生活条件。

　　（五）以交通、水利、电力为重点，加强基础设施建设。加快交通路网建设，主动服务金武、双营、永古高速公路建设，建成民勤、古浪客运中心和红沙岗货运中心，建设哈溪至古城等通乡公路。强化水利基础设施建设，全力推进杂木河毛藏寺水库建设和武威城区、大靖城区及重点工业园区供水工程。继续实施灌区续建配套与节水改造工程，开展红崖山等5座水库淤积治理，完成4座小型水库除险加固和六坝河、大靖河等河道防洪治理工程，抓好天然河道整治。实施好民勤县高效节水灌溉示范县、凉州区农业水价综合改革试点县、古浪县小型水利专项县等项目和天祝县山洪灾害防治非工程措施。争取天祝县进入牧区节水灌溉试点县实施范围。改造中低产田，建设高标准农田，新修梯田6万亩。加强电力基础设施建设，加快天祝宽沟、凉州雷台330千伏变电站建设进度，新开工建设古浪土门园区330千伏变电站及武南、打柴沟等5项增容改造项目，推进农网改造升级。加大城市建设投入力度，建成体育馆、博物馆等标志性建筑，基本建成新城区主干路网。加快老城区道路建设，改造城市棚户区1500户以上，完善城区供排水管网，推进城区天然气综合利用，抓好钟楼巷历史文化街区改造。加强集中供热建设和管理，适时启动城西集中供热工程。提升城市美化、绿化、亮化水平，着力打造环境优美、文明整洁、管理有序的城市形象。

　　（六）以物流业和旅游业为重点，积极发展现代服务业。着力发展现代物流业，加强与山西煤炭进出口公司的合作，推进武南煤炭集疏运中心建设。抓好黄羊众远公铁联运物流中心、武威综合物流园区和古浪国际物流园建设，培育现代物流企业和物流基地。实施万村千乡、双百市场、县乡集贸市场改造、农超对接等工程，建设改造专业批发市场、农村集贸市场、消费品市场、商品配送中心28个，着力提升商贸流通服务水平。围绕大宗农产品销售，健全完善农村运营体系，积极发展农村经济合作组织，加强储运设施建设，搞好产销对接服务，把利润空间最大限度地留给农民。大力发展旅游业，完成旅游规划编制工作，加快建设星级饭店，继续抓好旅游星级厕所建设，积极创建AAA级景区和年接待游客过30万人的景区，扶持建设5个旅游专业村，实现旅游接待人数310万人次。加大行业监管和培训力度，强化宣传推介，加强区域合作，大力开发特色旅游商品，不断提升旅游业发展水平。积极发展金融服务业，建立完善银政银企协调机制，推进全社会信用体系建设，积极扶持金融机构发展，引进更多金融机构设立分支机构，加快构建城乡融合发展、中小企业、城市建设融资平台，提高金融支持发展的能力。积极推进信息化建设，推进企业管理、农业生产经营、市场流通、交通运输信息化建设，发展电子政务、电子商务，提升工业园区、物流园区、农业产业化经营智能化建设水平。

　　（七）以完善民生保障体系为重点，协调发展各项社会事业。坚持教育优先发展，统筹调整城乡中小学布局结构，合理配置公共教育资源，继续实施中小学校舍安全工程，加强农村寄宿制学校建设。加快发展职业教育，支持兰州交通大学新能源与动力工程学院、甘肃威龙葡萄酒业专修学院、武威职业学院和古浪大靖等县区职教中心建设，尽快完成武威职业学院东西校区整体合并。实施学前教育推进工程，积极发展特殊教育。推进全民科学素质行动计划，把科技进步融入产业结构优化升级全过程，提高科技支撑能力。深化医药卫生体制改革，巩固国家基本药物制度，推进公立医院改革，优化医疗资源配置，实现新农合看病报销一卡通管理。加强基层医疗卫生服务体系建设，建成公共卫生综合服务楼、精神卫生专科医院、凉州区第三医院妇儿专科业务楼和天祝县藏医院业务楼，争取古浪中医院在大靖新城搬迁建设。扶持中医药和民族医药事业发展，努力创建全省中医先进市。加大统筹解决人口问题力度，完善利益导向政策体系，切实稳定低生育水平，提高出生人口素质。健全完善社会保障体系，扩大城镇职工养老、医疗、工伤、失业和生育保险范围，积极推进城乡居民养老保险工作，确保参保率达到90%以上。健全完善社会救助体系，及时解决城乡居民突发性、临时性困难。加大公积金归集扩面力度，加快保障性住房建设，有效抑制房价过快上涨。坚持以创业带动就业，用好小额担保贷款扶持政策，深入开展创办小企业、开发新岗位和培育“万名小老板”活动，确保高校毕业生就业率达到85%以上，城镇新增就业1.45万人。完善公共文化服务体系，发展公益性文化事业，加强文化人才队伍建设，推进文艺精品创作。扶持发展文化企业，推动经营性文化产业发展。加强对网吧、游戏厅等经营性文化娱乐场所的监管和整治，促进文化市场健康发展。加快广播电视“西新工程”、农村数字电影放映、乡镇文化站、村（社区）文化中心、农家书屋、基层体育场工程建设。落实党的民族宗教政策，维护藏区稳定；强化属地管理责任，促进宗教和顺。加强市场监管和调节，落实好“菜篮子”市长负责制，增加有效供给，努力保持物价基本稳定。强化食品药品安全监管，严厉打击制假售假行为。严格落实安全生产责任制，加强应急能力建设。扎实推进“六五”普法。加强信访工作，变上访为下访，将不稳定因素化解在基层和萌芽状态。加强社会管理综合治理，积极推进平安武威建设。加强全民国防教育、国防后备力量建设和人民防空工作，扩大双拥创建成果，争创全国双拥模范城。重视青少年教育成长，发展妇女儿童事业，推进扶残助残、敬老养老和慈善事业发展。继续做好外事侨务、地方志、档案、消防等工作。

　　（八）以创新体制机制为重点，着力提升改革开放水平。加大重点领域和关键环节的改革力度，探索建立适应跨越式发展的体制机制。推进行政管理体制改革，切实转变政府职能。深化行政审批制度改革，推进事业单位分类改革，提高行政效能。积极探索公共资源“市区合一”的运行体制，建立城镇、乡村、园区三位一体的管理模式，推进街道、社区改革。深化水权水价改革，探索水权交易模式，引导水权有序交易，推动水资源向低耗水、高效益行业优化配置。加快国有农林场改革步伐，切实做好马路滩、石羊河等国有农林场的改革工作，为重点企业发展、市内移民腾出空间。积极推进科技创新，围绕建设全省创新型城市、重点领域和产业发展，依托科研院所和企业，建立以企业为主体、市场为导向、产学研相结合的技术创新体系。加强高技能、实用型人才队伍建设，加大急需紧缺人才开发、引进力度。管好用活国土资源，坚持保护资源和保障发展并重，坚持集约用地，统筹利用城乡土地，建立投资、用地协调机制，完善土地资产收购、储备、开发和出让管理机制，用好石羊河流域重点治理压减耕地特殊政策，全力保障重点建设项目和工业用地。加快推进农村土地综合整治，加速农村土地承包经营权有序流转，提高农业规模化经营水平。

　　各位代表！群众的期盼就是政府努力的方向。明年我们将在完成省列实事办理任务的同时，拟投资20.27亿元，为民办好10件实事：1.解决18万农村人口安全饮水问题；2.新改建农村公路500公里；3.实施天马湖景观治理河道一期工程；4.实施海藏湖生态治理一期部分景观工程；5. 完成市综合客运枢纽中心主体工程；6.实施海拔2800米左右高深山区农牧民“下山入川”工程，完成移民1.7万人；7.新建廉租住房3902套、19.5万平方米；8.新增城区集中供热面积100万平方米；9.新改扩建校舍18万平方米，新改建幼儿园20所，新改建城乡融合发展核心区学校13所；10.开工建设新城区图书馆，完成影剧院主体工程。

　　四、加强政府自身建设

　　实施“三大战略”，推动经济社会跨越式发展，使命光荣，任务艰巨。我们必须按照“为民、务实、清廉、高效”的要求，努力建设人民满意的政府。

　　（一）加强理论武装，努力建设学习型政府。改革发展每推进一步，理论武装须先行一步。跨越式发展的目标任务，迫切要求我们强化理论武装，提高履职能力，建设一支高素质的公务员队伍。为此，必须把学习作为一种信仰、一种追求和一种境界，深入学习中国特色社会主义理论，学习党和国家各项方针政策，学习政治、经济、法律、历史、科技、管理等各方面知识，向书本学、向实践学、向群众学、向兄弟市州学，联系实际学、结合工作学、带着问题学，做到学以立德、学以增智、学以创新、学以创业，切实把学习的成果转化为推动发展的能力和本领。

　　（二）坚持依法行政，努力建设法治型政府。把法制建设作为行政权力科学高效运行的基础性工作，严格按照法定权限和程序履行职责、行使权力，自觉接受人大法律监督、政协民主监督及舆论监督、社会监督。全面落实依法行政实施纲要，坚持集体领导和分工负责制，健全完善民主决策、科学决策机制。深化政务公开和政府信息公开，加强电子政务建设，建立定期举办新闻发布会制度，推进权力公开透明运行。落实行政执法责任制，进一步规范行政执法行为，精减审批事项，减少审批环节。加强行政效能监察，实行行政问责制，完善绩效评估考核机制，坚决杜绝不作为、慢作为、乱作为现象，严厉查处失职、渎职行为，提高政府的公信力和执行力。

　　（三）改进工作作风，努力建设服务型政府。强化责任意识、服务意识，从群众最关心、发展最密切的困难和问题入手，重点在项目引进、立项、报批上跟踪搞好服务，在为创业者提供创业培训、信息咨询、融资贷款、人才引进上搞好服务，在打造公正透明的市场环境、宽松优越的创业环境、公平正义的法制环境上搞好服务。大兴求真务实之风，健全完善抓落实的领导机制和部门协调配合的工作机制，加强督促检查，强化层级责任，狠抓责任落实，减少会议文件，控制检查评比，树立强烈的时间观念和效率意识，雷厉风行，立说立行，坚决反对形式主义、官僚主义，力戒说大话、搞虚夸，聚精会神抓落实、促发展。

　　（四）不断解放思想，努力建设创新型政府。坚持用新理念指导实践，用新思维破解难题，用新标准推动发展，尊重创造，鼓励创新，营造充满活力的社会发展环境。围绕思想、制度、政策等深层次问题，抓住扩大投资、培植财源、促进就业、稳定物价等重点领域和关键环节，深入开展调查研究，积极探索新思路、新办法、新举措，以改革创新的办法破解发展中遇到的难题。完善保障跨越式发展的体制机制，破除各种陈规陋习，从不断变化的形势中捕捉和把握发展机遇，在困难交织的环境中发现和培育有利因素，创造性地开展工作。

　　（五）加强反腐倡廉，努力建设廉洁型政府。全面落实党风廉政建设责任制，健全完善惩治和预防腐败体系，加强对重要岗位、关键环节的监督和制约，规范权力运行，加强领导干部经济责任审计，更加有效地预防腐败。完善政府重大投资项目公示制和责任追究制，规范土地招拍挂、建设工程招投标、政府采购和产权交易等行为，严厉查处各种违纪违法案件，坚决纠正损害群众利益的各种不正之风。牢固树立过紧日子的思想，按照节俭、高效、廉洁的原则，深入推动节约型机关建设，积极推进公务消费和公务接待制度改革，大力削减行政成本，勤俭节约办事业。

　　各位代表！实干收获希望,拼搏创造未来。我们有信心、有决心、也有能力担负起人民赋予的光荣使命。让我们在市委的坚强领导下，在市人大、市政协和社会各界的监督支持下，以只争朝夕的精神状态、攻坚克难的昂扬锐气、求真务实的工作作风，开拓创新，奋发进取，为全面实现武威跨越式发展的目标任务而努力奋斗！
